# Unacylated Ghrelin Protects Against Age‐Related Loss of Muscle Mass and Contractile Dysfunction in Skeletal Muscle

**DOI:** 10.1111/acel.14323

**Published:** 2024-09-02

**Authors:** Hyunyoung Kim, Rojina Ranjit, Dennis R. Claflin, Constantin Georgescu, Jonathan D. Wren, Susan V. Brooks, Benjamin F. Miller, Bumsoo Ahn

**Affiliations:** ^1^ Department of Internal Medicine Wake Forest University School of Medicine Winston‐Salem North Carolina USA; ^2^ Aging and Metabolism Research Program Oklahoma Medical Research Foundation Oklahoma City Oklahoma USA; ^3^ Department of Biochemistry University of Oklahoma Health Sciences Center Oklahoma City Oklahoma USA; ^4^ Department of Biomedical Engineering University of Michigan Ann Arbor Michigan USA; ^5^ Department of Molecular and Integrative Physiology University of Michigan Ann Arbor Michigan USA; ^6^ Genes and Human Disease Research Program Oklahoma Medical Research Foundation Oklahoma City Oklahoma USA; ^7^ Oklahoma City VA Medical Center Oklahoma City Oklahoma USA

**Keywords:** loss of muscle mass, mitochondria, neurogenic atrophy, neuromuscular junction, protein synthesis and degradation, sarcopenia, unacylated ghrelin

## Abstract

Sarcopenia, the progressive loss of muscle mass and function, universally affects older adults and is closely associated with frailty and reduced quality of life. Despite the inevitable consequences of sarcopenia and its relevance to healthspan, no pharmacological therapies are currently available. Ghrelin is a gut‐released hormone that increases appetite and body weight through acylation. Acylated ghrelin activates its receptor, growth hormone secretagogue receptor 1a (GHSR1a), in the brain by binding to it. Studies have demonstrated that acyl and unacylated ghrelin (UnAG) both have protective effects against acute pathological conditions independent of receptor activation. Here, we investigated the long‐term effects of UnAG in age‐associated muscle atrophy and contractile dysfunction in mice. Four‐month‐old and 18‐month‐old mice were subjected to either UnAG or control treatment for 10 months. UnAG did not affect food consumption or body weight. Gastrocnemius and quadriceps muscle weights were reduced by 20%–30% with age, which was partially protected against by UnAG. Specific force, force per cross‐sectional area, measured in isolated extensor digitorum longus muscle was diminished by 30% in old mice; however, UnAG prevented the loss of specific force. UnAG also protected from decreases in mitochondrial respiration and increases in hydrogen peroxide generation of skeletal muscle of old mice. Results of bulk mRNA‐seq analysis and our contractile function data show that UnAG reversed neuromuscular junction impairment that occurs with age. Collectively, our data revealed the direct role of UnAG in mitigating sarcopenia in mice, independent of food consumption or body weight, implicating UnAG treatment as a potential therapy against sarcopenia.

## Graphical abstract

Unacylated ghrelin treatment mitigates age‐related decline in muscle mass, strength, neuromuscular coupling, and mitochondrial function.

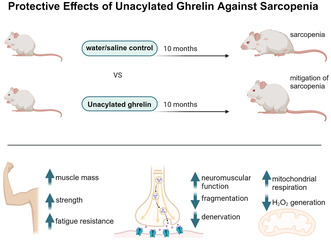

**Abbreviations**
AchR: acetylcholine receptor
AmA: antimycinA
Asc: ascorbate
AUR: Amplex UltraRed
D_2_O: deuterium oxide
EDL: extensor digitorum longus
FWHM: full width at half‐maximum
G/M: glutamate/malate
GHSR1a: growth hormone secretagogue receptor 1a
H_2_O_2_
: hydrogen peroxide
HRP: horseradish peroxidase
ICT: intracellular calcium transient
IGF‐1: Insulin‐like growth hormone factor‐1
IPA: ingenuity pathway analysis
Lo: optimal length
NMJ: neuromuscular junction
OCR: oxygen consumption rate
PCA: principal component analysis
ROS: reactive oxygen species
Rot: rotenone
SEM: standard error of means
sFo: specific force
SOD: superoxide dismutase
Sod1KO: superoxide dismutase 1KO
Suc: succinate
UnAG: unacylated ghrelin

## 1 INTRODUCTION

Sarcopenia is the decline of skeletal muscle mass and strength that universally occurs independent of disease incidence. Sarcopenia significantly impacts older adults, often resulting in a loss of mobility and independence, thereby affecting quality of life and health span (Larsson et al., 2019). Key factors contributing to sarcopenia include mitochondrial dysfunction (Coen et al., 2019), oxidative stress (Ahn et al., 2019), and neuromuscular junction disruption (Jang & Van Remmen, 2011). Because of the projected growth of the geriatric population, the needs for therapeutic interventions against age‐associated sarcopenia will rapidly and consistently grow in the coming decades. Resistance training is known to increase muscle mass and protect against sarcopenia (Cannataro et al., 2022; Laurin et al., 2019); however, no pharmacological treatments are currently available.

Ghrelin is a peptide hormone consisting of 28 amino acids that is primarily produced in the stomach. A small subset of ghrelin undergoes acylation at the serine 3 site, upon which acyl ghrelin binds to growth hormone secretagogue receptor 1a (GHSR1a) in the brain increasing appetite (Kojima et al., 1999). Most of the circulating ghrelin—90%–95%, depending on energy balance—remains in the unacylated form (Porporato et al., 2013), which does not bind to the receptors in the brain. Although both acyl and unacylated ghrelin (UnAG) have direct effects on peripheral tissues and organelles independent of biological activities of GHSR1a (Filigheddu et al., 2007; Gortan Cappellari et al., 2017; Ku et al., 2016; Porporato et al., 2013; Rossetti et al., 2017; Sheriff et al., 2012; Wagner et al., 2017), acyl ghrelin increases adiposity, which can negatively impact muscle due to insulin resistance, altered metabolism, and intracellular signaling detrimental to force generation and muscle growth (Picard et al., 2012). We previously reported sarcopenic effects and adipogenesis in response to a GHSR1a receptor agonist in wildtype and Sod1KO mice, a mouse model of elevated oxidative stress (Ranjit et al., 2022). Furthermore, a recent study by Kaiser et al. demonstrated life‐extending effects of the GHSR1a receptor agonist when the mice were pair‐fed with control animals (Kaiser et al., 2023). Collectively, although the literature indicates promising effects of ghrelin on peripheral tissues and aging, the biological effects of ghrelin independent of the GHSR1a receptor remain unclear.

Several studies have demonstrated the direct effects of UnAG in peripheral tissues, including neurons and skeletal muscle (Agosti et al., 2020; Gortan Cappellari et al., 2017; Ku et al., 2016; Porporato et al., 2013; Wagner et al., 2017). Cultured neuronal cells co‐incubated with UnAG were protected against cell death induced by mitochondrial inhibitors (Wagner et al., 2017), and results of an in vivo study showed that UnAG can protect against stroke‐induced brain injury (Ku et al., 2016). In skeletal muscle, UnAG increases satellite cell function and enhances myogenesis in cultured myoblasts (Filigheddu et al., 2007; Porporato et al., 2013). Myofiber growth and hypertrophy were reported after acute pathological conditions that led to atrophy and weakness (Porporato et al., 2013; Togliatto et al., 2013). Given the direct impact of UnAG on neurons and muscle cells, we hypothesized that UnAG will protect against the loss of muscle mass and contractile dysfunction by enhancing protein balance and mitochondria in sarcopenia.

## 2 METHODS

### 2.1 Animal care

Four‐month‐ and 18‐month‐old female and male C57Bl/6N mice were obtained from the National Institute of Aging. All mice were caged in pathogen‐free conditions and provided with free access to standard chow and water and maintained on a 12‐h light/dark cycle. Both female and male mice were kept in a temperature‐controlled room (21°C ± 1°C). The Institutional Animal Care and Use Committee at Oklahoma Medical Research Foundation approved all experiments and procedures.

### 2.2 UnAG delivery via pump implantation

**FIGURE 1:**
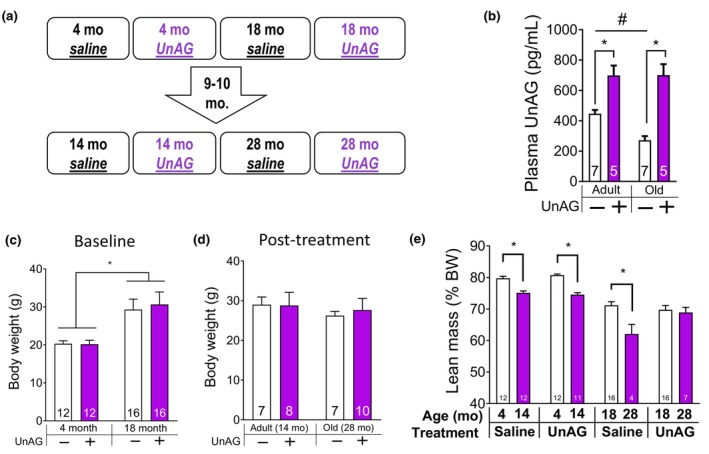
UnAG delivery via osmotic pump increased plasma UnAG levels and normalized the decreased lean body mass in old mice at the study endpoint. (a) Study design demonstrates 10 months delivery of UnAG via osmotic pump in female mice. (b) After treatment, plasma UnAG levels are elevated in adult and old mice treated with UnAG relative to control mice. (c) Body weights at baseline, at 4 and 14 months old. (d) Body weights at the post‐treatment endpoint, at 14 (adult) and 28 months of age (old). (e) Percentage lean body mass relative to body mass before and after UnAG treatment. *n* = 4–16. Numbers within each bar denote the number of mice included for specific groups. Two‐Way ANOVA (age × treatment) followed by Tukey post hoc analyses were used to determine statistical significance. *p* < 0.05. *Treatment effect. #Age effect. UnAG, unacylated ghrelin.

**FIGURE 2:**
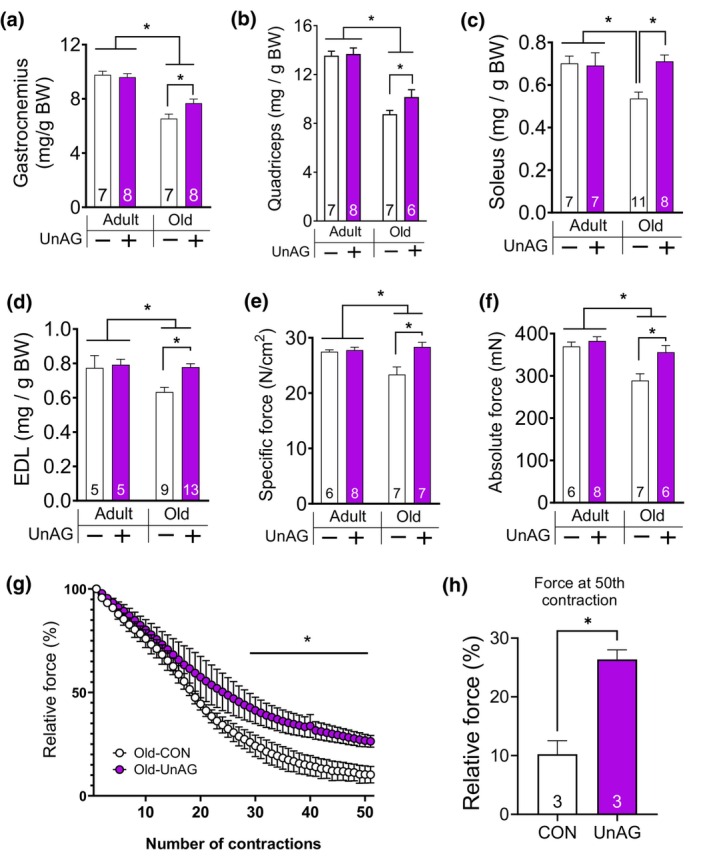
UnAG treatment increased muscle mass, strength and fatigue resistance in old female mice. All mice included in this analysis had been treated via osmotic pump. After UnAG or saline treatment of adult and old female mice, (a) gastrocnemius, (b) quadriceps, (c) soleus, and (d) EDL muscle masses in adult and old mice. (e) Absolute force (mN) from isolated EDL muscle. (f) Specific force, force per cross‐sectional area, using isolated EDL muscle. Two‐way ANOVA (age × treatment) followed by Tukey post hoc tests were used to determine statistical significance (a–f). (g) Relative force changes (as percentage of initial force) during a fatigue protocol. Two‐way ANOVA followed by Bonferroni post hoc analyses were used to determine statistical significance. (h) Percentage of initial force at select timepoints. *p < 0.05. Numbers within each bar denote the number of mice. EDL, extensor digitorum longus.

UnAG, purchased from Anaspec®, was dissolved in saline and loaded in osmotic pumps (2004, Alzet®). We performed surgeries on female mice to implant osmotic pumps to achieve continuous release at a rate of 0.11 μL/h UnAG, which delivers approximately 100 μg UnAG/kg body weight each day. This concentration was shown to be protective against muscle wasting in acute pathological conditions and insults (Agosti et al., 2020; Porporato et al., 2013; Ranjit et al., 2022; Rossetti et al., 2017). Control groups received saline through the osmotic pumps. We started the treatment in 4‐ and 18‐month‐old mice (before significant changes in muscle mass and force occur) and treated the mice until 14 and 28 months (for approximately 10 months) (Figure 1a). As each pump lasts for 2 months, we replaced the pumps four times during the course of the treatment. At the study endpoint, we sacrificed the animals, drew blood from their vena cava, and separated plasma samples to determine UnAG levels in circulation. We used an ELISA kit from Bertin Bioreagent and followed the assay protocols provided by the supplier. The results generated from UnAG delivery via pump implantation were for female mice only and are presented in Figures 1 and 2. The figures for studies involving female mice only are color coded in purple for clarification.

### 2.3 Body composition

Animals treated using osmotic pumps (saline or UnAG) were used for determining body composition. Quantitative magnetic resonance imaging was used to determine the percentages of body fat and lean mass (Lustgarten et al., 2009). Live mice were placed into a thin‐walled plastic cylinder (4.7 cm internal diameter, 0.15 cm thick), where they were free to turn around but were limited to approximately 4 cm vertical movements by a plastic insert. The plastic cylinder containing the live mice was then placed into the qMRI machine (EchoMRI, Echo Medical Systems, Houston, TX, USA) to measure both lean and fat mass. Once the measurements were completed, the mice were returned to their home cage. The methods were used for female mice.

### 2.4 In vitro contractile force assessment

We assessed force generation of extensor digitorum longus (EDL) in vitro in mice treated via osmotic pump as previously described (Roberts et al., 2013). Mice were sacrificed using gaseous carbon dioxide, and one EDL muscle was immediately excised and prepared for functional assays in a bicarbonate‐buffered solution gassed with a mixture of 95% O_2_ and 5% CO_2_ at room temperature. We placed the EDL muscle in an organ bath containing bicarbonate‐buffered solution at room temperature and determined the length that induces maximal twitch force, that is, optimal length (*L*_O_). Muscles were allowed 10 min of thermal equilibration at 32°C. Measurements of force‐frequency were then initiated. In all electrical stimulations, a supramaximal current (600–800 mA) of 0.25 ms pulse duration was delivered through a stimulator (Aurora Scientific Inc., 701C). Five minutes after completing the force‐frequency protocol, the EDL muscle was stimulated to fatigue with isometric contractions (pulse frequency 150 Hz, train duration 500 ms, train rate 0.25 Hz). All data were recorded and analyzed using commercial software (DMC and DMA, Aurora Scientific). Specific force (N/cm^2^) was calculated by force and estimated fiber cross‐sectional area (in cm^2^), which is based on fiber length/muscle length ratio of EDL, as published previously (Brooks & Faulkner, 1988). The methods were used for female mice.

### 2.5 Delivery of UnAG in drinking water

**FIGURE 3:**
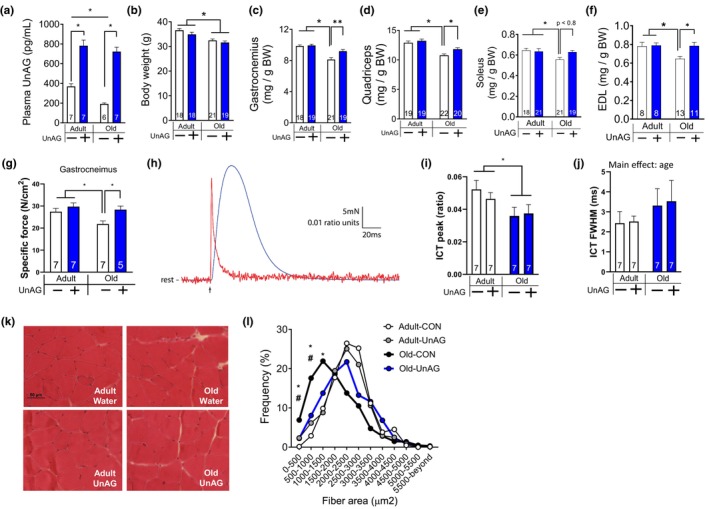
UnAG increased muscle quantity and quality without affecting body weights in male mice. All mice included in this analysis had been treated via drinking water. (a) Delivering UnAG via drinking water increased plasma UnAG levels in adult and old male mice. (b) Body weights of adult (14 months) and old (28 months) mice. Muscle mass was normalized to body weight (mg/g) for (c) gastrocnemius, (d) quadriceps, (e) soleus, and (f) EDL muscles. (g) Gastrocnemius muscle force generation when directly stimulated by electrode. Specific force, force per estimated cross‐sectional area. (h) Representative lumbrical muscle force (blue) and mag‐fura‐2 fluorescence ratio (red) responses to twitch excitation. Individual responses were elicited by a single stimulus pulse (0.2 ms duration) and 16 recordings were averaged for each of two excitation wavelengths (344 nm, 375 nm). The averages were used to form the mag‐fura‐2 ratio shown (344/375). Arrow indicates the time at which the stimulation was delivered. (i) Peak of the intracellular calcium transient (ICT) indicates the height of mag‐fura‐2 fluorescence ratio relative to resting level and (j) ICT FWHM (full width at half‐maximum) indicates the time during which mag‐fura‐2 fluorescence ratio remains at or above its half maximum level. (k) Representative histological images of hemotoxylin and eosin staining from gastrocnemius muscle. (l) Frequency histogram based on myofiber areas. *Represents statistical difference between adult control and old control. #Represents statistical difference between old control‐treated and old UnAG‐treated mice. *n* = 4–5. Two‐way ANOVA (age × treatment) followed by Tukey post hoc analyses were used to determine statistical significance. *p < 0.05. Data are mean ± SEM. Numbers within each bar denote the number of mice.

**FIGURE 4:**
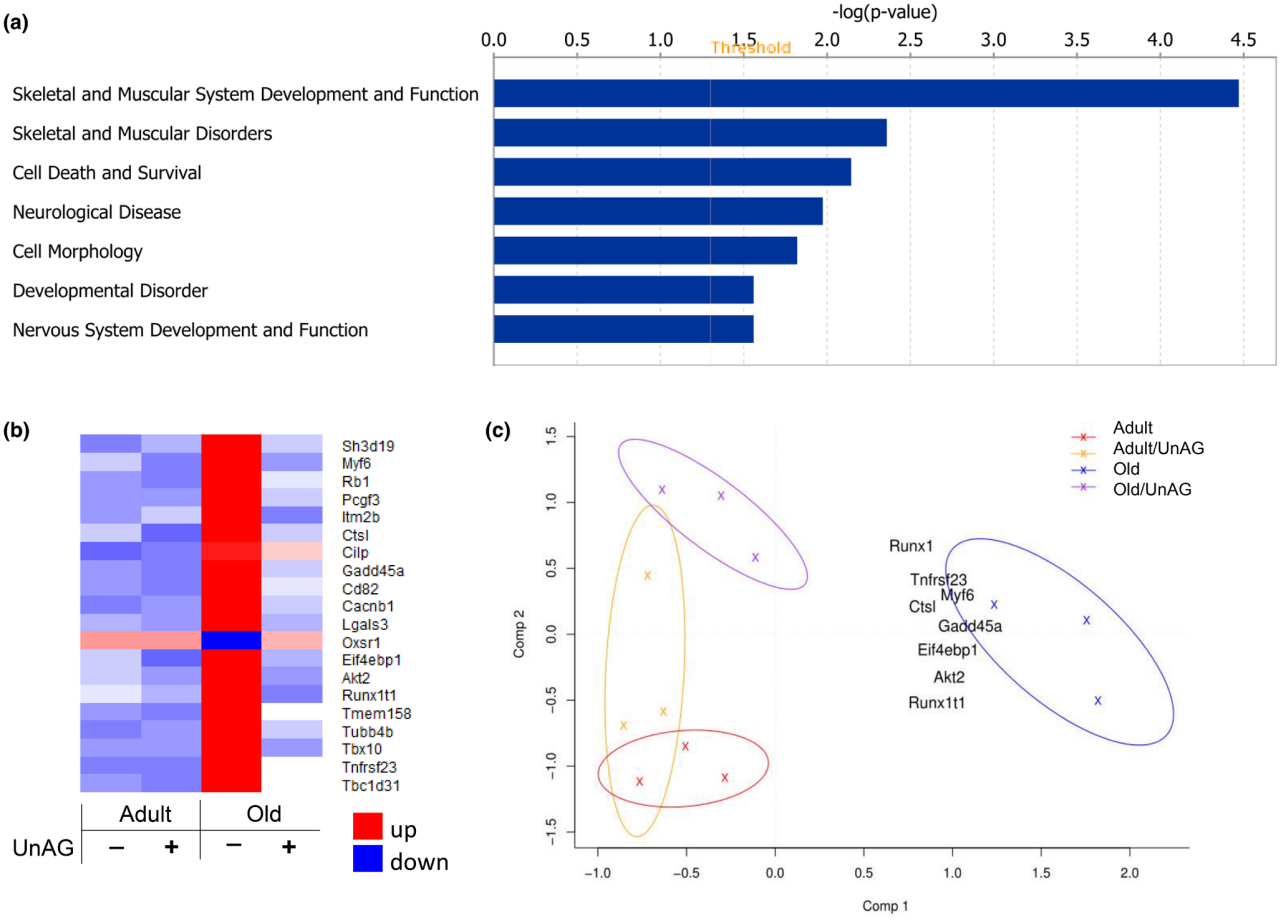
Bulk mRNAseq analysis from gastrocnemius muscle indicates downstream effects induced by UnAG. All mice included in this analysis were males that had been treated via drinking water. (a) Ingenuity pathway analysis (IPA) showing the top 7 canonical pathways differentially regulated by UnAG in old mice relative to control mice. (b) A heat map showing top 20 genes differentially regulated by old but shifted back to ‘adult’ level by UnAG. Genes are ordered by *q*‐values (adjusted *p*‐value for false discovery rate). (c) Principal component (PC) plot shows several differentially regulated genes in old mice, but UnAG normalized genes similar to adult mice. Benjamini–Hochberg multiple testing was applied to determine the differentially expressed transcripts.

**FIGURE 5:**
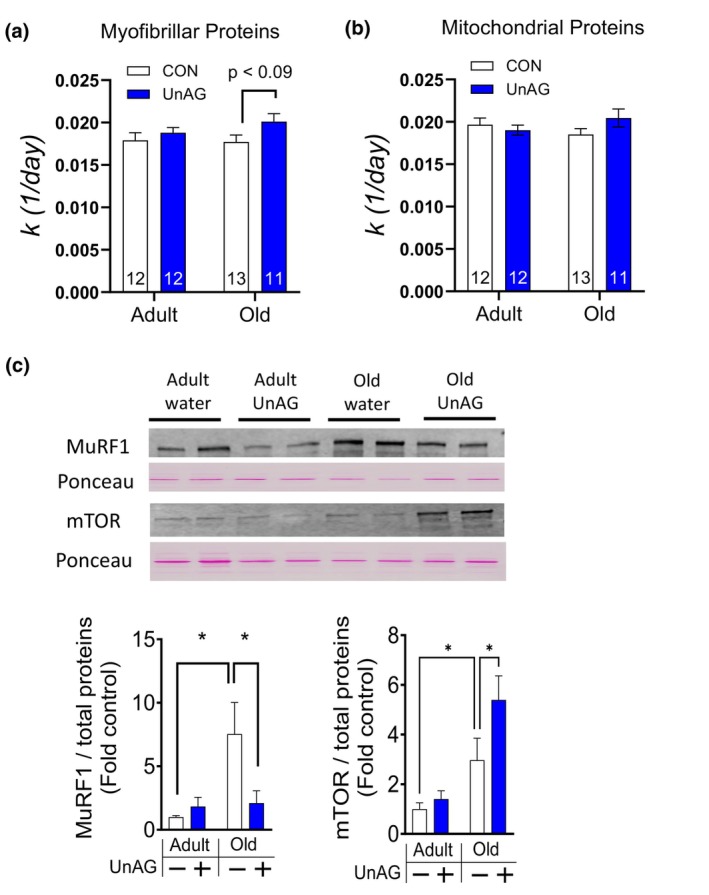
Protein synthesis rate in vivo determined by deuterium oxide treatment. All mice included in this analysis were males that had been treated via drinking water. Protein synthesis rates of (a) myofibrillar protein fraction and (b) mitochondrial fraction. Numbers within each bar denote the number of mice. (c) Western blot analyses showing MuRF1, MAFbx, and mTOR expression levels in gastrocnemius homogenates. *n* = 4. Two‐way ANOVA (age × treatment) followed by Tukey post hoc analyses were used to determine statistical significance. *n* = 4. *p < 0.05.

**FIGURE 6:**
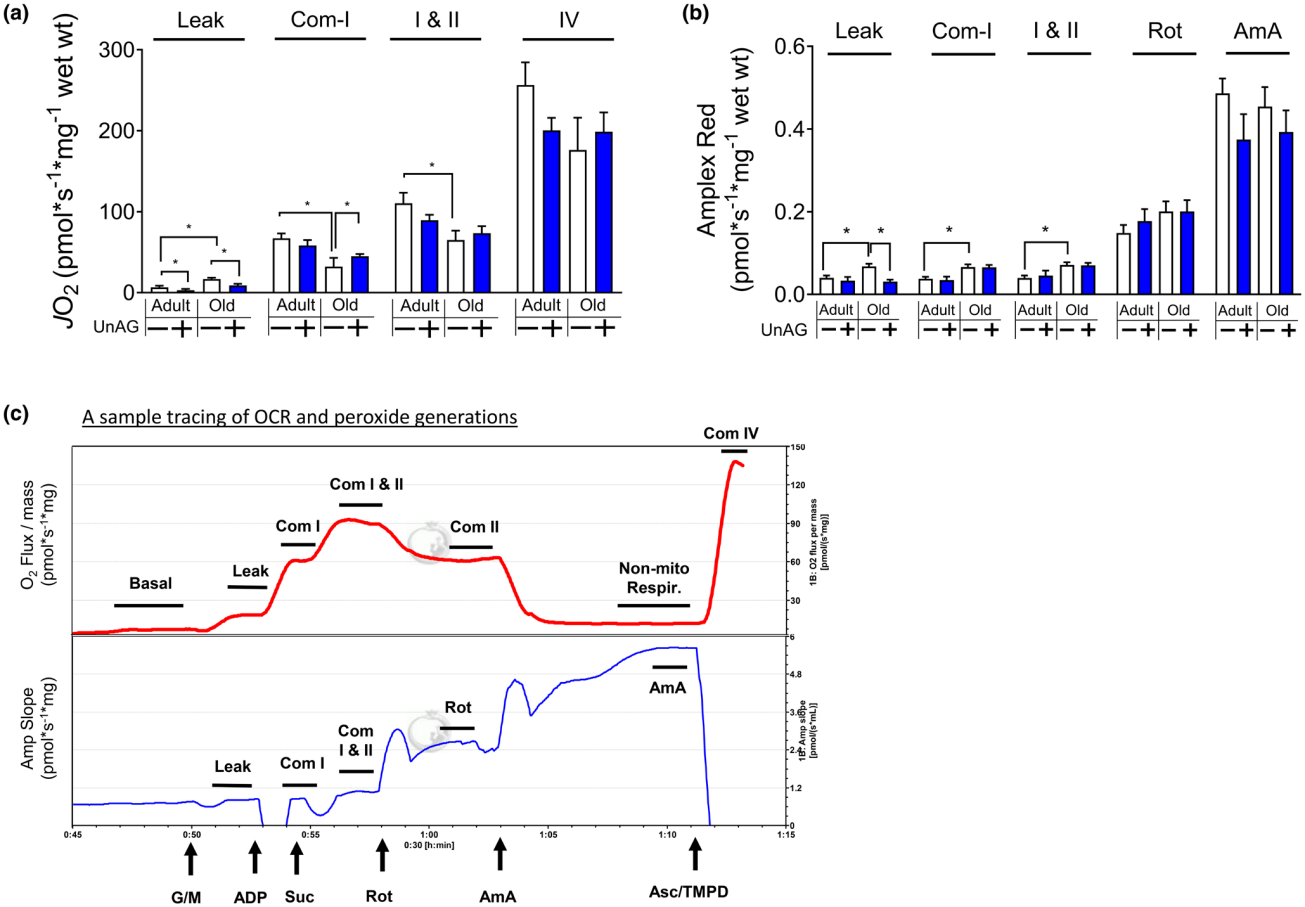
UnAG improves mitochondrial respiration and ROS generation rates in skeletal muscle of old mice. All mice included in this analysis were males that had been treated via drinking water. (a) Mitochondrial respiration is measured using permeabilized myofibers from red gastrocnemius muscle. Substrates were added to activate individual complexes of the mitochondria. Glutamate and malate (G/M) for Complex I, succinate (Suc) for Complex II, and ascorbate/TMPD (Asc/TMPD) for Complex IV. ADP was added for state III respiration. Rotenone (Rot) was added to inhibit Complex I and antimycin A was added to inhibit mitochondrial respiration. (b) Hydrogen peroxide generation rate was simultaneously determined in the presence of Amplex UltraRed (AUR), horseradish peroxidase (HRP) and superoxide dismutase (SOD). *n* = 7–8. (c) Sample tracing of OCR and hydrogen peroxide generation rate data. Two‐way ANOVA (age × treatment) followed by Tukey post hoc analyses were used to determine statistical significance. *p < 0.05.

**FIGURE 7:**
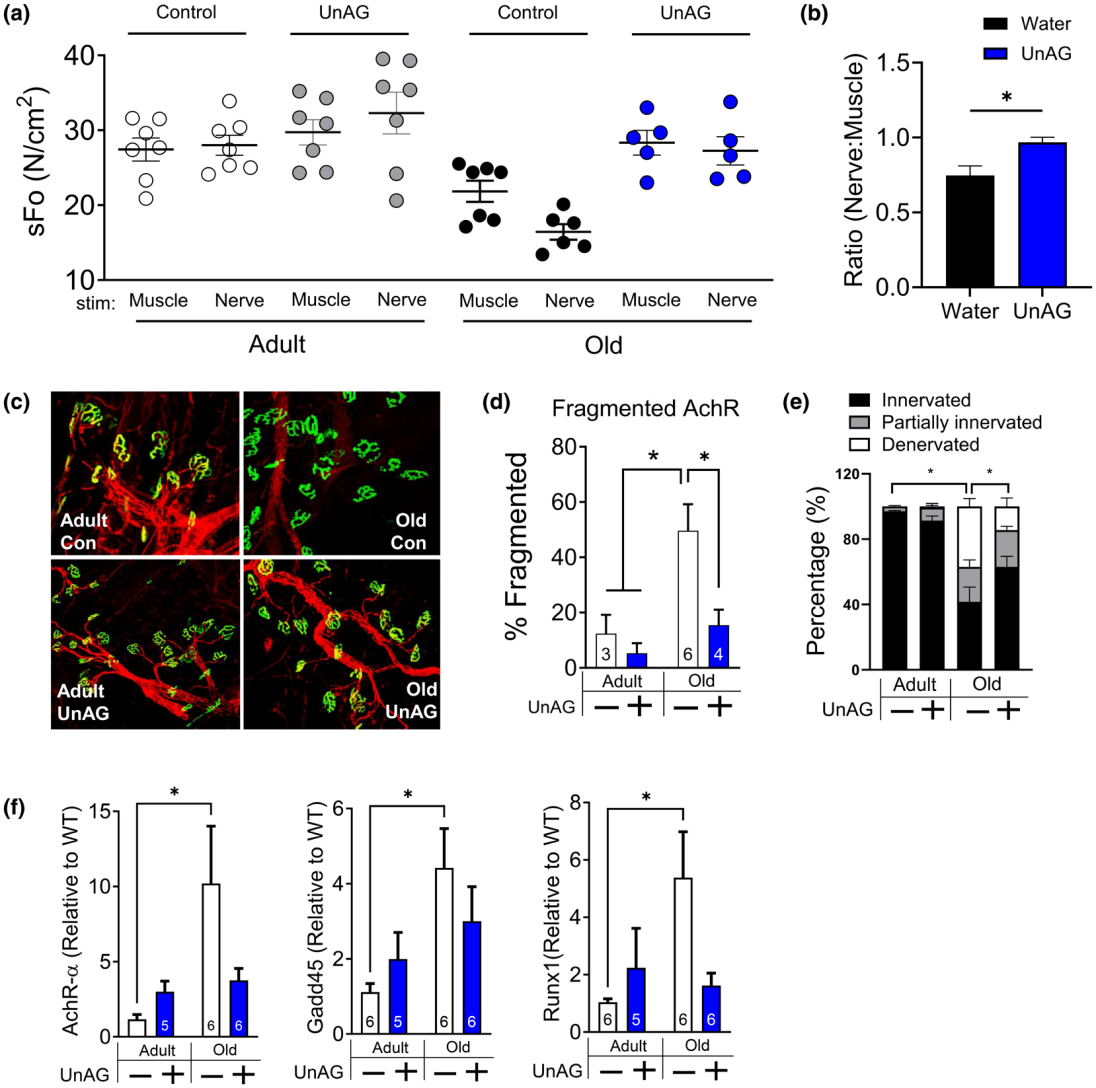
UnAG improved neuromuscular function and NMJ morphology in gastrocnemius muscle of old mice. All mice included in this analysis were males that had been treated via drinking water. (a) Specific force generation by gastrocnemius muscles of control and UnAG treated mice elicited by nerve or direct muscle stimulation. (b) Ratio of force induced by nerve and muscle stimulation. Sciatic nerve‐stimulated force is lower than muscle stimulated force (i.e., force deficit) in gastrocnemius muscle of old mice, but UnAG normalizes the force deficit. (c) Histological images of neuromuscular junction (NMJ). Green indicates acetylcholine receptors (AchR), whereas red indicates motor neuron. Yellow indicates innervation. (d) Percentage of fragmented AchR. *n* = 3–6. (e) Percentage of denervated NMJ. *n* = 3–6. (f) mRNA levels of genes elevated in response to denervation and aging. Two‐way ANOVA (age × treatment) followed by Tukey post hoc analyses were used to determine statistical significance. *p < 0.05. Numbers within each bar denote the number of mice. AchR, acetylcholine receptor; stim, stimulation.

Osmotic pump delivery requires several surgeries under anesthesia because each pump lasts 6 weeks maximum. Even though the procedure is brief, multiple surgeries can induce inflammation, which may impact skeletal muscle. To rule out any potential confounding effects of multiple surgeries and anesthesia, we treated a separate cohort of male mice with UnAG via their drinking water. On the basis of the daily water consumption of our animals (5.3 mL/day, Figure S1a in Data S1), we dissolved UnAG compounds in drinking water to ensure that the mice in UnAG group received approximately 100 μg/kg/day; control groups received drinking water only. We found that UnAG was stable at least for 1 week in drinking water. Thus, we replaced the water bottles weekly for both UnAG‐treated and control groups. Mice were treated for 10 months. Our previous data showed that UnAG treatment does not change their water or food consumption (Ranjit et al., 2022). The results generated from UnAG delivery via drinking water were for male mice only and are presented in Figures 3, 4, 5, 6, 7. The figures representing male mice only are color‐coded in blue for clarification.

### 2.6 In situ and whole tissue force assessment

To investigate changes in neuromuscular function, we compared forces elicited by nerve versus direct muscle stimulation of contraction in an in situ contractile function preparation, as described previously (Ahn et al., 2019). Briefly, gastrocnemius muscle was dissected from surrounding muscle and connective tissue with great care to avoid damaging the neighboring nerves and/or blood vessels during the dissection. The hind limb was securely tied to a fixed post with 4‐0 monofilament nylon suture at the knee, and the foot was clamped to the platform. The distal tendon of the gastrocnemius muscle was then tied to the lever arm of a servomotor (Aurora Scientific). Muscle length was adjusted to the optimal length (*L*o) at which twitch force was maximal. With the muscle held at *L*o, 300‐ms trains of stimulus pulses were applied at increasing stimulation frequencies until maximum isometric tetanic force (Po) was achieved. Then, we then repeated the experiment with the gastrocnemius muscle stimulated directly to bypass the neuromuscular junction and assess force generation.

The methods and instrumentation for measuring the contractility of the lumbrical muscles have been described in detail previously (Claflin & Brooks, 2008). Measurements were made using a custom‐built chamber with a quartz floor to allow excitation of the mag‐fura‐2 fluorescent dye with ultraviolet light. Experimental temperature was 25°C and acquisition and analysis were performed using custom software developed in LabVIEW (National Instruments).

### 2.7 Intracellular calcium transients

Experiments to determine the dynamic calcium responses to twitch stimuli were performed on lumbrical muscles by loading the low‐affinity calcium indicator mag‐fura‐2 AM (10 μM; ThermoFisher) for 30 min at 25°C. Pluronic F‐127 (0.01% *w*/*v*; ThermoFisher) was used as a dispersing agent. Mag‐fura‐2 fluorescence was excited at 344 nm and 375 nm (bandwidth 10 nm) and responses were passed through a 510 nm emission filter (bandwidth 40 nm) before entering a photomultiplier tube (Hamamatsu model R1527) operated in analog mode. The excitation source was a 75 W xenon lamp, and wavelengths were selected using a diffraction grating monochromator (PTI, model DeltaRAM). The experimental sequence consisted of first recording twitch force and mag‐fura‐2 fluorescence during excitation at 344 nm, waiting 20 s, and then recording twitch force and mag‐fura‐2 fluorescence during excitation at 375 nm. The alternating sequence was repeated until a total of 16 responses had been recorded at each excitation wavelength. Pre‐loading backgrounds were then subtracted from all fluorescence records and the 16 responses at each excitation wavelength were averaged. Finally, the ratio of the averaged mag‐fura‐2 fluorescence intensities (344/375) was taken as an undistorted representation of the rapid intracellular changes in calcium concentration that give rise to a twitch contraction (Konishi et al., 1991). Additional details on the techniques and apparatus used to measure lumbrical muscle contractile properties and fluorescence have been described previously (Claflin & Brooks, 2008).

### 2.8 Fiber permeabilization

Preparation for skeletal muscle fiber permeabilization was performed as previously described (Ahn et al., 2018). Briefly, a small piece (approximately 3–5 mg) of red gastrocnemius muscle was carefully dissected, and we separated fibers in ice‐cold buffer X containing 7.23 mM K_2_EGTA, 2.77 mM CaK_2_EGTA, 20 mM imidazole, 0.5 mM DTT, 20 mM taurine, 5.7 mM ATP, 14.3 mM phosphocreatine (PCr), 6.56 mM MgCl_2_‐6H_2_O, and 50 mM K‐MES (pH 7.1). The muscle bundle was permeabilized in saponin solution (30 ug/mL) for 30 min, followed by three 5‐min washes in ice‐cold wash buffer Z containing 105 mM K‐MES, 30 mM KCl, 10 mM K_2_HPO_4_, 5 mM MgCl_2_‐6H_2_O, 0.5 mg/mL bovine serum albumin (BSA), and 0.1 mM EGTA (pH 7.1).

### 2.9 Simultaneous measurement of respiration and hydrogen peroxide production

Oxygen consumption rate (OCR) and the rate of mitochondrial hydrogen peroxide production were simultaneously determined using the Oxygraph‐2k (O2k, OROBOROS Instruments, Innsbruck, Austria) by a previously described method with minor modifications, as previously described (Ahn et al., 2018). OCR was determined using an oxygen sensor, and rates of hydrogen peroxide generation were determined using the O2k‐Fluo LED2‐Module Fluorescence‐Sensor Green. Measurements were performed on permeabilized fibers in buffer Z media at 37°C containing 10 μM Amplex® UltraRed (Molecular Probes, Eugene, OR, USA), 1 U/mL horseradish peroxidase (HRP), and 25 μM blebbistatin. HRP catalyzes the reaction between hydrogen peroxide and Amplex UltraRed to produce the fluorescent resorufin (excitation: 565 nm; emission: 600 nM). The fluorescent signal was converted into nanomolar H_2_O_2_ via a standard curve established on each day of experiments. Sample tracing for H_2_O_2_ standard is shown in Figure 6d. Background resorufin production was subtracted from each measurement. Rates of respiration and H_2_O_2_ production were determined using sequential additions of substrates and inhibitors as follows: glutamate (10 mM), malate (2 mM), ADP (5 mM), succinate (10 mM), rotenone (1 μM), antimycin A (1 μM), and TMPD (0.5 mM) immediately followed by ascorbate (5 mM). All respiration measurements were normalized to antimycin A to account for non‐mitochondrial oxygen consumption. Data for both OCR and rates of hydrogen peroxide generation were normalized by milligrams of muscle bundle wet weights.

### 2.10 Histology

Gastrocnemius muscles were frozen in liquid nitrogen‐cooled isopentane. We made 5 μm cross‐sections in the mid‐belly of the muscle using a microcryotome (−20°C) and dried the sections for 1 h. The sections were rehydrated and stained with hematoxylin and eosin. Hematoxylin and eosin sections were scanned, and the images were used to determine cross‐sectional area and to determine fiber areas in gastrocnemius muscles using Image J. We analyzed 4–5 slides for each mouse. Each slide contained 80–140 myofibers. Myofibers deeper within the tissue were used for quantifications.

### 2.11 Staining of neuromuscular junction

We dissected fresh gastrocnemius muscle, cleaned connective tissue, and cut it into small pieces in cold PBS. We fixed the tissue in 10% STUmol (Poly Scientific R&D, Bay Shore, NY, USA) in ddH_2_O for 1 h on a rocker, washed three times for 5 min and permeabilized in 2% Triton X‐100 in PBS for 30 min. After blocking overnight at 4°C in 5% NGS, 4% BSA, and 1% Triton X‐100 in PBS, the samples were incubated with primary antibodies for 24–48 h. The samples were washed six times for 30 min each in PBS and incubated overnight with a secondary antibody and bungarotoxin conjugated to a fluorophore. The samples were washed again in PBS (six times, 30 min for each wash), blotted with a moistened kimwipe, mounted onto Superfrost Plus Slides (VWR) with EMS Glycerol Fluoromount with PPD anti‐fading agent mounting medium (EMS, Hatfield, PA, USA), and cover‐slipped for imaging. We analyzed 30–50 images for each mouse.

### 2.12 Immunoblot

Western blots were performed using SDS–PAGE electrophoresis. Gastrocnemius tissue lysates were prepared in a Ripa buffer containing 50 mM Tris‐Cl (pH 7.4), 1 mM EDTA, 0.5 mM EGTA, 1% Triton X‐100, 0.1% sodium deoxycholate, 0.1% SDS, and 140 mM NaCl. Equal amounts of protein samples (10–20 μg) were loaded on 10%–12.5% SDS–PAGE gels (casting system from Bio‐Rad) with 1× Tris/Glycine/SDS buffer. Proteins were transferred onto PVDF or nitrocellulose membranes overnight at 4°C with wet‐transfer equipment. Blots were imaged using Odyssey DLx imaging system and quantified by Image J and Empiria Studio® softwares (LI‐COR, Lincoln, NE, USA). Signal densities of target proteins were normalized by total proteins. Table S1a in Data S1 lists the primary antibodies used in this study for western blots.

### 2.13 Time course labeling by deuterium oxide

We used deuterium oxide (D_2_O) labeling to measure protein synthesis rates. Mice were labeled at multiple time points as previously described (Reid et al., 2020). In the last month of the UnAG or control treatment, animals were divided into subgroups that received D_2_O for 0, 7, 14, and 28 days at sacrifice (*n* = 3 each group at each timepoint, *n* = 12 total per group). Labeling of deuterium oxide (D_2_O) was initiated with a priming intraperitoneal bolus injection of 99.9% (Sigma‐Aldrich), which was followed by 8% D_2_O‐enriched drinking water. D_2_O administration was staggered so that all mice were sacrificed at exactly 4 weeks of treatment.

### 2.14 Fractionation of muscle homogenates and determination of protein synthesis rate

Protein synthesis was determined using our established methods (Miller et al., 2019, 2020). Briefly, approximately 30–50 mg of skeletal muscle tissue was homogenized 1:20 in isolation buffer (100 mM KCl, 40 mM Tris HCl, 10 mM Tris Base, 5 mM MgCl_2_, 1 mM EDTA, 1 mM ATP, pH = 7.6) with phosphatase and protease inhibitors (HALT, Thermo Fisher Scientific) using a bead homogenizer (Next Advance, Inc., Averill Park, NY, USA). After homogenization, subcellular fractions were isolated via differential centrifugation to obtain myofibrillar and mitochondrial protein fractions. Protein fractions were derivatized for analysis of deuterium enrichment in alanine using an Agilent 7890A GC coupled to an Agilent 5975C MS (Lawrence et al., 2020). To determine the precursor pool enrichment, plasma samples were prepared for analysis of deuterium enrichment on a liquid water isotope analyzer (Los Gatos Research, Los Gatos, CA, USA) using 0%–12% deuterium standards (Groennebaek et al., 2018). The precursor enrichment of alanine was then adjusted by mass isotopomer distribution analysis (Hellerstein & Neese, 1999). The deuterium enrichments of both the protein (product) and the precursor were used to calculate: fraction new = E_product_/E_precursor_, in which the E_product_ is the enrichment (E) of protein‐bound alanine and E_precursor_ is the calculated maximum alanine enrichment from equilibration of the body water pool. The fraction new data were then plotted across the timepoints, and curves were fit to the data using one‐phase associations (Reid et al., 2020; Wolff et al., 2020) using Graphpad Prism 10 (GraphPad Software, San Diego, CA, USA). The software calculates rate parameter (*k*, 1/day), which reflects the protein synthetic rate. We constrained the plateau (*p*) to 1.0, assuming the protein pool fully renews.

### 2.15 Bulk RNA sequencing and transcriptomics analysis

TRIzol (Invitrogen, CA, USA) was used to isolate messenger RNA (mRNA) from approximately 20 mg of gastrocnemius muscles from four groups, adult and old male mice ± UnAG treatment. Three samples from each group were used for sequencing. The samples were prepared and processed by the Clinical Genomics Center at Oklahoma Medical Research Foundation (OMRF; omrf.org/researchfaculty/core‐facilities/next‐generation‐sequencing/). TruSeq Stranded mRNA Library Kit (Illumina) was used for library preparation, and the samples were sequenced on Illumina NextSeq 500. The results were then processed and analyzed by OMRF Genomics and Data Science group. Raw sequencing reads (in a FASTQ format) were trimmed of residual adaptor sequences using Scythe software. Low‐quality bases at the beginning or the end of sequencing reads were removed using sickle; then the quality of remaining reads was confirmed with FastQC. Trimmed sequencing reads were aligned to the Mus musculus genome reference (GRCm38/mm10) using STAR v2.4.0 h. Gene‐level read counts were determined using HTSeq v0.5.3p9 with the GENCODE Release M10 (GRCm38) annotation. Total reads from each sample ranged from 20 to 30 M. Read‐count normalization and differentially expressed analyses were performed using the edgeR package from Bioconductor. Expression values normalized with the voom function were analyzed for differential expression using the standard functions of the limma package. Three hundred and sixty‐six genes differentially expressed in old mice, reversing to younger mice expression levels after gremlin treatment, were identified by collapsing mid‐aged controls and both mid‐aged and old treated mice into a single group, and contrasting it to old mice. Since the *t*‐statistic considers the difference between group averages relative to within‐group variation, a strong *t*‐statistic would indicate both strong difference between old mice versus the other groups, and similar expression levels in the mid‐aged and treated mice. Moderate *t*‐test *p*‐values were adjusted for multiple testing using the false discovery rate (FDR) method and FDR < 0.05 was used to filter for significant differences. Ingenuity Pathway Analysis (IPA, QIAGEN, Redwood City, CA, USA) was used to identify and explore significant pathways, functional sets and gene networks interactively.

### 2.16 qRT‐PCR

Total RNA was extracted from gastrocnemius using TRIzol reagent (Invitrogen, Carlsbad, CA, USA). Equal amounts of extracted RNA (1 μg) were converted to first strand cDNA using a cDNA synthesis kit (Bio‐Rad, Herculus, CA, USA). 5 ng of the cDNA samples was amplified using primers for AchRα, GADD45α, Runx1, and Sarcolipin. Primer sequences are found in Table S1 in Data S1. Real time PCR (RT‐PCR) was performed in Quant Studio 6 (Applied Biosystems, Foster City, CA, USA). The ΔΔCt method was used to calculate relative mRNA expression.

### 2.17 Statistical analyses

Graphpad Prism 10 was used for graphing and statistical analyses. After confirming normality, unpaired two‐tailed *t*‐tests were used to compare two groups, while a two‐way ANOVA was used to assess the effects of ghrelin treatment and age with post hoc multiple comparisons tests. Principal component exploratory analysis (PCA) of sample similarity, and biplot generation were carried out with specific functions from stats package in R. Multidimensional 95% confidence intervals were added to the biplot using draw.ellipse function from plotrix package.

## 3 RESULTS

### 3.1 UnAG treatment increases plasma levels of UnAG at the end point and protects against the loss of lean body mass in old female mice

Female C57Bl/6N wildtype mice, 4‐month‐olds and 18‐month‐olds, were treated with UnAG via osmotic pump for 10 months (Figure 1a). Plasma UnAG level was significantly lower in old mice than in young mice, but the UnAG‐treated group had higher terminal plasma UnAG levels than the control (Figure 1b). The animal body weights remained similar between control and UnAG‐treated groups (Figure 1c,d). Percentage lean body mass significantly decreased over 10 months in both age group controls, but 18‐month‐old mice treated with UnAG maintained lean body mass over 10 months (Figure 1e). Notably, the preservation of lean body mass was only seen in 18‐month‐old mice; the 4‐month‐old mice treated with UnAG lost lean body mass over 10 months.

### 3.2 UnAG treatment increases muscle mass, strength, and fatigue resistance in old female mice

Consistent with the changes in the lean body mass, gastrocnemius and quadriceps weights were reduced by 20%–30% with age, declines that were partially mitigated by UnAG (Figure 2a,b). Relatively smaller lower limb muscles, including slow‐twitch dominant (e.g., soleus) and fast‐twitch dominant muscles (e.g., EDL), also showed age‐associated decreases in muscle mass, which were protected by UnAG (Figure 2c,d). To test the impact of UnAG on muscle quality, we tested contractile properties of fast twitch EDL muscles. The maximum isometric specific force, force per cross‐sectional area (sFo), was significantly decreased in old mice compared to young, consistent with previous reports (Xu et al., 2022). Muscle mass was partially preserved by UnAG, whereas muscles of old mice treated with UnAG showed no decline in sFo (Figure 2e,f). To further investigate the role of UnAG in muscle metabolism and fatigability during muscle contractions, EDL muscles were subject to repetitive electrical stimulations (i.e., fatigue protocol). Rate of force decline was attenuated in muscles treated with UnAG in old animals (Figure 2g,h), indicating the protective effects of UnAG against fatigue in old mice. UnAG did not alter the rate of force decline in young mice (Figure S1 in Data S1).

### 3.3 UnAG increases muscle quantity and quality without affecting body weights in old male mice

To rule out potential confounding effects of anesthesia and inflammation as a result of repeated pump implantation surgeries, we delivered UnAG via drinking water for 10 months to 4‐month‐ and 18‐month‐old male mice. Pilot experiments had shown that male mice drink 5–6 mL water each day (mean 5.3 mL/day) and similar amounts in mice 4–27 months old (Figure S2 in Data S1). We found that male mice that received UnAG‐dissolved drinking water had increased plasma UnAG levels at the endpoint (Figure 3a). Interestingly, plasma UnAG levels were lower in males than in females at 14‐months old but decreased to a similar level in both sexes by 28 months old (Figure S3 in Data S1). Body weight was lower for old mice than for adult (Figure 3b). Relative muscle mass of lower limb muscles decreased in old mice but improved by UnAG treatment (Figure 3c–f). In situ contractile function via direct stimulation on gastrocnemius was substantially decreased in old mice but normalized by UnAG (Figure 3g). Improvement in force generation can be achieved by multiple factors, including, but not limited to, calcium sensitivity, calcium release, and calcium uptake mechanisms (Kim & Heckman, 2023). Thus, we monitored intracellular calcium transients (ICTs) in real time using lumbrical muscles. We found approximately 30% decreases in the height of the peak of the ICT in old muscle relative to adult muscle (Figure 3h,i), which was not affected by UnAG. Age had a significant main effect in the duration of ICT full width at half‐maximum by approximately 40% in old mice; however, post hoc analyses revealed no statistical differences (Figure 3j). In histological analyses of myofiber areas, frequency distribution analysis demonstrated a rightward shift by UnAG in old mice (Figure 4k,l), indicating hypertrophy of myofibers by UnAG.

### 3.4 RNAseq data indicate downstream effects of UnAG

To investigate downstream pathways that are up‐ or down‐regulated by UnAG, we performed an unbiased approach of bulk mRNA sequencing and found pathways that are differentially regulated. Top seven canonical pathways included skeletal and muscular system development and function, muscular disorder, cell death and survival, neurological disorders, cell morphology, developmental disorder, and nervous system development and function (Figure 4a). Using heat map analysis, we identified the top 20 genes differentially expressed in response to UnAG. The changes in the genes induced by aging were upregulated, except oxidative stress responsive kinase1 gene (Oxsr1) (Figure 4b). The PCA plot (Figure 4c) revealed that several differentially expressed genes with age were reversed by UnAG, which included transcriptional markers of denervation (i.e., Gadd45a, Runx1) and genes involved in protein synthesis (Eif4EBP1) and proteolysis (cathepsin L, Ctsl). The transcription markers of denervation were elevated in other pathological conditions, including denervation model and mouse model of redox‐dependent sarcopenia (Ahn et al., 2022; Ebert et al., 2012).

### 3.5 UnAG impacts skeletal muscle protein degradation and synthesis of older mice

In a further attempt to elucidate the mechanisms by which UnAG increases muscle quantity, we treated a subset of animals with stable isotope deuterium oxide and determined the rate of protein synthesis using fractions of skeletal muscle tissue. UnAG has been shown to directly increase myoblast differentiation and fusion formation by activating pathways involved in myogenesis in cultured myoblasts (Filigheddu et al., 2007). UnAG also increased mitochondrial OCR while downregulating reactive oxygen species generation from the mitochondria (Cappellari et al., 2016; Rossetti et al., 2017). Thus, we fractionated gastrocnemius into myofibrillar and mitochondrial parts using previously established procedures (Robinson et al., 2011). We found a trend of increased protein synthesis rates from myofibrillar fraction (*p* < 0.09; Figure 5a). Our data support in vivo effects of UnAG on muscle growth that was observed in the results from cultured myotubes (Filigheddu et al., 2007; Porporato et al., 2013). We further explored pathways involved in protein synthesis and degradation, including mTOR, MuRF1 and MAFbx and found upregulation of mTOR proteins and downregulation of MuRF1 (Figure 5c) in response to UnAG; MAFbx protein expression were unchanged (Figure S4 in Data S1).

### 3.6 UnAG improves mitochondrial respiration while decreasing reactive oxygen species generation rates

We assessed mitochondrial OCR and hydrogen peroxide generation rate to determine the role of UnAG in mitochondria. In old animals, we found that leak respiration, or oxygen consumption driven by proton leak in mitochondrial matrix, was elevated. This increase in leak respiration contributes to oxidative damage to muscle tissues (Jastroch et al., 2010). However, UnAG attenuated the increased leak respiration in old mice. We found that OCR of old mice was substantially downregulated, consistent with results from a published study (Xu et al., 2022). UnAG increased Complex I‐mediated OCR, whereas Complex I and II‐induced OCR remained unchanged by UnAG treatment in either young or old mice (Figure 6a). Increased hydrogen peroxide generations in old mice can result in oxidative damage in skeletal muscle, which can impair contractile function (Ahn et al., 2018, 2019; Czyzowskaa et al., 2023). Results of our assessment revealed that UnAG normalized the increased respiration in the leak state in old mce (Figure 6a). Likewise, rate of hydrogen peroxide generation rate was decreased by UnAG (Figure 6b). The experiments were performed within 30–40 min, as shown in sample tracing (Figure 6c).

### 3.7 UnAG improves neuromuscular function and NMJ morphology

NMJ disruption has been reported to occur as a result of aging‐ and inactivity‐induced sarcopenia, both in humans (Campbell et al., 1973; Oda, 1984) and other mammals (Ahn et al., 2018; Hashizume & Kanda, 1995; Xu et al., 2022). In our study, compared to direct muscle‐stimulated force, nerve stimulation‐induced force generation by muscles of old animals was significantly lower (Figure 7a,b), which is consistent with the literature (Ahn et al., 2018; Xu et al., 2022). Notably, old mice treated with UnAG did not exhibit differences between nerve‐ and direct muscle‐stimulated force, demonstrating the protective effects of UnAG on neuromuscular function. Furthermore, we observed that UnAG protected against fragmentation and loss of innervation in gastrocnemius muscle of old mice (Figure 7c–e). Our findings are supported by our transcriptomic data, in which transcriptional markers of denervation and aging (i.e., Gadd45a and Runx1) were upregulated in old mice, but this effect was normalized by UnAG (Figure 4b,c).

## 4 DISCUSSION

The goal of our study was to test the therapeutic potential of UnAG in age‐associated sarcopenia. The long‐term treatment of UnAG rescued old mice from the loss of lean body mass and muscle atrophy, which were associated with downregulation of ubiquitin–proteasome pathways. UnAG increased force generating capacity of the muscle (i.e., specific force). Our transcriptomic analyses further revealed differentially regulated genes by UnAG, including transcriptional markers of denervation. UnAG improved mitochondrial respiration and decreases in hydrogen peroxide generation rate, consistent with the improvement in fatigue resistance. Our results collectively demonstrate that UnAG improved skeletal muscle mass and quality during aging.

UnAG did not affect food consumption, body weight, or adiposity in the current study. Previously, when we treated wildtype C57Bl/6N mice with the orexigenic acyl ghrelin receptor mimetic, fat mass increased with a decrease in lean body mass, muscle mass, and strength (Ranjit et al., 2022). A previous study also addressed the adverse effects of adipogenesis and muscle weakness by acyl ghrelin receptor agonist and its limitation as treatment against sarcopenia (Wu et al., 2020). We further demonstrated that one or two months of treatment with GHSR1a receptor agonist surprisingly decreased anabolic pathways by acyl ghrelin, including insulin‐like growth hormone factor‐1 (IGF‐1) and proteins involved in myogenesis (Ranjit et al., 2022). Furthermore, results of a recent study demonstrated that a lifelong stimulation of ghrelin GHSR1a receptor increased lifespan of C57Bl/6J male mice when the mice were pair‐fed to control animals (Kaiser et al., 2023). Both acyl ghrelin and UnAG have been shown to have direct effects on peripheral tissues, including, but not limited to, skeletal muscle (Filigheddu et al., 2007; Porporato et al., 2013), neurons (Ku et al., 2016; Raimondo et al., 2013), bone (Delhanty et al., 2006; Kim et al., 2005), and the heart (Baldanzi et al., 2002). Collectively, these studies beg the question of whether ghrelin without activation of its GHSR1a receptor can protect from the neurogenic atrophy, mitochondrial dysfunction, and loss of strength that are associated with age.

We explored downstream effects of UnAG in skeletal muscle using global RNA sequencing. Interestingly, the results of our heat map analysis revealed that the top 20 genes that are differentially expressed in aged mice and normalized by UnAG were mostly upregulated, except for Oxsr1. Oxsr1 is thought to play an important role in salt transport, the immune response, and oxidative stress (Gagnon & Delpire, 2012; Li et al., 2004). The functional significance of Oxsr1 has not been previously explored in skeletal muscle or in aging, but single nucleotide polymorphisms of Oxsr1 were associated with exacerbation of the risk of asthma and altered smooth muscle cell function (Kim et al., 2022). Further, the PCA plot demonstrates that several genes differentially regulated in response to aging are reversed by UnAG, including transcriptional markers of denervation (i.e., Gadd45a, Runx1). These transcriptional markers are also shown to be elevated in various pathological conditions and diseases resulting in muscle wasting and weakness in rodents (Chai et al., 2011) and humans (Oda, 1984).

Our data demonstrate that UnAG protects against sarcopenia by modulating several downstream factors. UnAG increases protein synthesis while downregulating protein degradation pathways mediated by E3 ligases MuRF1 and MAFBx. UnAG improves mitochondrial respiration and protects against NMJ disruption in old mice. Other groups demonstrated protective effects of UnAG on acute pathological conditions that result in muscle atrophy and weakness. For example, Portorato et al. demonstrated that transgenic mice with UnAG overexpression (Myh6/Ghrl, 50‐fold elevation in circulation compared to control) were protected against fasting‐ and denervation‐induced atrophy (Porporato et al., 2013). Although Porporato et al. found downregulation of atrogin1 in Myh6/Ghrl mice, our data demonstrate downregulation of MuRF1 by UnAG treatment. The discrepancy might be caused by the different levels of increase by two different treatment strategies, or different assays used for assessment of downregulation pathways. We determined protein expression by western blot, whereas Porporato et al. measured gene expression by qRT PCR. UnAG has been shown to protect against high fat diet‐ and chronic kidney disease‐induced muscle atrophy by improving mitochondrial respiration and hydrogen peroxide generation (Cappellari et al., 2016; Gortan Cappellari et al., 2017). These findings are consistent with our data in sarcopenia. Finally, the role of UnAG on neuromuscular junction disruption and neuromuscular coupling are first reported by our group, to our knowledge.

Neuronal deficits and neuromuscular junction disruption are important contributors and presumably upstream to sarcopenia (Chai et al., 2011; Ivannikov & Van Remmen, 2015;Oda, 1984; Tomlinson & Irving, 1977). NMJ degeneration, retraction of motor neurons and elevated muscle mitochondrial ROS generations are common observations in aged humans (Oda, 1984; Tomlinson & Irving, 1977) and rodents (Chai et al., 2011; Ivannikov & Van Remmen, 2015). Studies demonstrated that muscle and neuronal oxidative stress cause NMJ disruption, denervation, and contractile dysfunctions (Ahn et al., 2019, 2022; Xu et al., 2021). In addition to the improvement in force generation in isolated EDL muscle, UnAG also preserved nerve‐stimulated force output, indicating its effects on both neurons and muscle. These functional data are consistent with the normalization of acetylcholine receptor (AchR) area and reduction of fragmentation in old mice. Furthermore, markers of denervation are also normalized by UnAG (AchR, GaDD45, Runx1, and Sarcolipin), supporting the global RNAseq data. Considering the protective effects of UnAG on neuronal cells (Ku et al., 2016; Lim et al., 2011; Wagner et al., 2017), we believe that the protection that UnAG offers against sarcopenia is, at least in part, derived from its impacts on neurons.

Lean body mass decreased over the 10‐month treatment in both young and old control mice, which was fully prevented by UnAG in old mice. We also found that gastrocnemius and quadriceps muscle masses were significantly higher at the older age in UnAG‐treated group than in the control group, which is consistent with a previous study (Agosti et al., 2020). In our study, UnAG increased fiber size; however, Agosti et al. did not observe changes in cross‐sectional area. This discrepancy may be due to the treatment method and duration, as well as the mice age. Indeed, the differences in muscle mass between UnAG and control mice are substantially greater in our study (40%–50% vs. 10%–15%) (Agosti et al., 2020). To identify the driving mechanisms of greater muscle masses in UnAG‐treated mice, we directly measured in vivo protein synthesis rates using the stable isotope deuterium oxide. Skeletal muscle tissues were fractionated into myofibrillar and mitochondrial proteins. Protein synthesis rates of myofibrillar fraction trended towards higher levels with UnAG treatment in the old age group, whereas mitochondrial fractions remained unchanged by the treatment. Similarly, Sheriff et al. also showed that UnAG increases protein synthesis rates in cultured cells using [3H]‐tyrosine isotope (Sheriff et al., 2012). To investigate protein degradation pathways, we performed immunoblot assays and found that the E3 ligase MuRF1 was significantly elevated in old skeletal muscle, which was normalized by UnAG. Consistent with our data, UnAG decreased MuRF1 expression in cultured C_2_C_12_ myotubes treated with dexamethasone (Porporato et al., 2013). Collectively, our data show that UnAG affects both protein synthesis and degradation pathways and increases lean body mass and muscle quantity in old mice.

Decreased mitochondrial bioenergetics and early onset of fatigue contribute to mobility decline in older adults, and findings from the Baltimore Longitudinal Study of Aging demonstrated that mitochondrial energetics predicts mobility decline in older adults (Tian et al., 2022). Notably, UnAG has been shown to increase muscle mitochondrial respiration in a Complex I‐specific manner (Cappellari et al., 2016), which is consistent with our findings. We further demonstrate that these effects on mitochondrial function are reflected in muscle function as evidenced by the improved ability of muscles from UnAG‐treated old mice maintaining force during repetitive contractions.

Excess reactive oxygen species and oxidative stress are known to play important roles in force generation. For example, oxidative protein modifications modulate force output in skeletal muscle (Ahn et al., 2019, 2022; Moopanar & Allen, 2006). Consistent with the literature, rates of hydrogen peroxide generation found in the present study were increased in old skeletal muscle at leak state (Kumaran et al., 2005; Tonkonogi et al., 2003). Our respirometry data further support and show higher levels of leak respiration in old compared with adult mice. The significance of proton leak on redox balance, aging, and neurodegenerative diseases has been reported previously (Marcinek et al., 2005). Importantly, long‐term treatment with UnAG prevented the changes in mitochondrial hydrogen peroxide generation rates in skeletal muscles with age. Thus, we conclude that the preservation of muscle strength was, at least in part, due to normalized redox homeostasis by UnAG. Additional regulating factors for loss of force generation with age are reductions to calcium release, uptake, and sensitivity with age (Xu et al., 2022). We tested intracellular calcium release and uptake kinetics in response to UnAG treatment and found that they were unaffected. Under normal conditions, intracellular calcium reaches concentrations that are sufficient to saturate the regulatory protein binding sites very early in a tetanic contraction (Baylor & Hollingworth, 2012), so maximum tetanic force is relatively insensitive to small variations in tetanic calcium levels. Thus, our data indicate that UnAG normalized tetanic force deficit in old skeletal muscle, which is, at least in part, associated with mitochondrial hydrogen peroxide and oxidative stress.

Previous research investigated the effects of UnAG on male or female rodents (Agosti et al., 2020; Cappellari et al., 2016; Gortan Cappellari et al., 2017; Porporato et al., 2013; Ranjit et al., 2022; Togliatto et al., 2013). To our knowledge, this is the first study to investigate UnAG effects on both sexes. We demonstrate that UnAG was effective for male and female mice, although a limitation of our study is that our molecular experiments were mainly conducted on male mice. We cannot determine whether UnAG activates similar or different biological pathways in female mice. Interestingly, we found that plasma UnAG levels between male and female mice were different at 14 months of age (Figure S3 in Data S1). The levels decreased to a similar level for both sexes at approximately 28 months of age. The sex‐ and age‐dependent changes in UnAG levels may involve the changes of X/A‐like cells in the stomach and small intestine, which produce most of the circulating ghrelin (Ibrahim Abdalla, 2015). Additionally, it is also possible that enzymes involved in cleavage of preproghrelin have reduced concentration or activity with age and sex, but no such findings have been reported to date to our knowledge.

In summary, our data demonstrate that UnAG counteracts the loss of muscle mass and strength in old mice. These protections were associated with normalized mitochondrial respiration, ROS generations, and neuromuscular coupling in skeletal muscle of old mice. Our findings are highly relevant to human aging and sarcopenia because ghrelin and its mimetics have been used in humans and reported in the literature (Allas et al., 2018; Tong et al., 2014). Several clinical trials are underway (e.g., NCT04377126, NCT03358355, and NCT04768803) and our findings provide important mechanistic insights regarding the cellular and molecular basis for the protective effects. UnAG holds promise for preventing against and treating sarcopenia for improved mobility and healthy lifespan in older adults.

## AUTHOR CONTRIBUTIONS

B.A. conceptualized and designed the project; H.K., R.R., and D.R.C. performed the experiments. B.A., H.K., D.R.C., analyzed the data. C.G. and J.D.W. analyzed RNA sequencing data. B.A. interpreted the data. B.A., B.F.M., J.D.W., and S.V.B. wrote and edited the manuscript.

## CONFLICT OF INTEREST STATEMENT

The authors declare no conflicts of interest.

## Supporting information


Data S1.
**FIGURE S1.** (a) Relative force (in percent of initial force) with repeated contractions during fatigue protocol in female adult mice treated with saline (sal) or UnAG. (b) Percentage of initial force at 50th contraction (*n* = 3). Two‐way ANOVA with Tukey’s post hoc analysis was used to determine differences between groups. Statistical significance was determined at *p* < 0.05; no difference was detected. Data are mean ± SEM. UnAG, unacylated ghrelin.
**FIGURE S2.** The amount of water consumed each day (mL/day) by male mice aged 4–27 months (*n* = 46). Water consumption did not correlate with age.
**FIGURE S3.** Plasma UnAG levels of control‐treated male and female mice. PBS was delivered via osmotic pump for female mice, and water was delivered via drinking water for male mice. Two‐way ANOVA (age × sex) followed by Tukey’s post hoc analysis was used to determine the differences between groups. Statistical significance was determined at *p* < 0.05. No difference was detected. Data are mean ± SEM. UnAG, unacylated ghrelin.
**FIGURE S4.** Western blot results showing MAFbx expression levels in gastrocnemius homogenates of male mice. *n* = 4. Two‐way ANOVA (age × treatment) was used to determine statistical significance; no difference was found.
**FIGURE S5.** Example of tracing for the hydrogen peroxide standard assay, which was performed each day of experiments. Each arrow indicates addition of 0.1 μM hydrogen peroxide. Inlet shows standard curve between hydrogen peroxide and Amp Raw [V].
**TABLE S1.** Lists of primary antibodies (a) and primer sequences (b) used to determine protein expression and gene expression.

## Data Availability

The data that support the findings of this study are available from the corresponding author upon reasonable request. The data are not publicly available due to privacy or ethical restrictions.
