# Supplementary material for: Unacylated Ghrelin Protects Against Age‐Related Loss of Muscle Mass and Contractile Dysfunction in Skeletal Muscle
Source: Aging Cell. 2024 Sep 2;23(12):e14323. doi: 10.1111/acel.14323 (PMC11634730; doi:10.1111/acel.14323)
Supplement: Supplementary file 1 — Data S1. FIGURE S1. (a) Relative force (in percent of initial force) with repeated contractions during fatigue protocol in female adult mice treated with saline (sal) or UnAG. (b) Percentage of initial force at 50th contraction (n = 3). Two‐way ANOVA with Tukey’s post hoc analysis was used to determine differences between groups. Statistical significance was determined at p < 0.05; no difference was detected. Data are mean ± SEM. UnAG, unacylated ghrelin. FIGURE S2. The amount of water consumed each day (mL/day) by male mice aged 4–27 months (n = 46). Water consumption did not correlate with age. FIGURE S3. Plasma UnAG levels of control‐treated male and female mice. PBS was delivered via osmotic pump for female mice, and water was delivered via drinking water for male mice. Two‐way ANOVA (age × sex) followed by Tukey’s post hoc analysis was used to determine the differences between groups. Statistical significance was determined at p < 0.05. No difference was detected. Data are mean ± SEM. UnAG, unacylated ghrelin. FIGURE S4. Western blot results showing MAFbx expression levels in gastrocnemius homogenates of male mice. n = 4. Two‐way ANOVA (age × treatment) was used to determine statistical significance; no difference was found. FIGURE S5. Example of tracing for the hydrogen peroxide standard assay, which was performed each day of experiments. Each arrow indicates addition of 0.1 μM hydrogen peroxide. Inlet shows standard curve between hydrogen peroxide and Amp Raw [V]. TABLE S1. Lists of primary antibodies (a) and primer sequences (b) used to determine protein expression and gene expression. [file ACEL-23-e14323-s001.pdf]

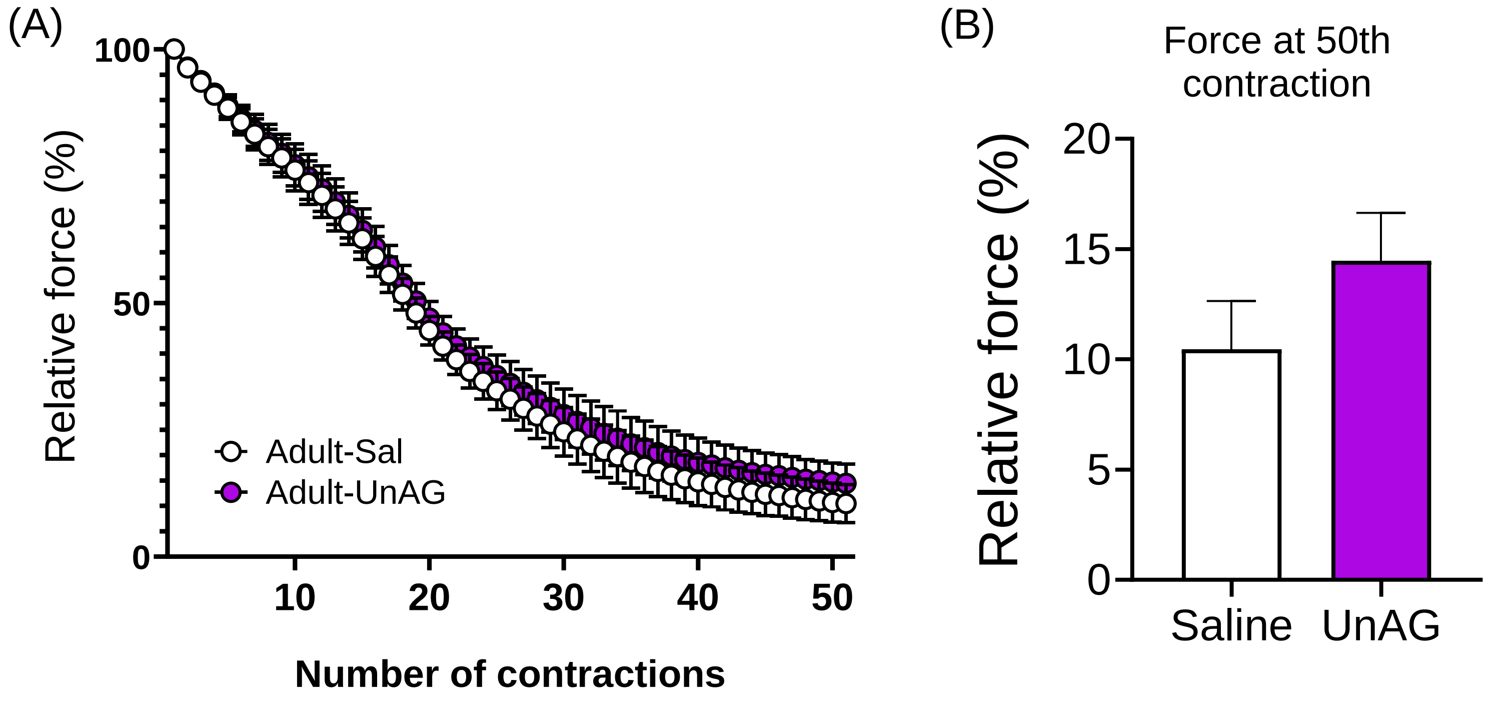

Supplemental Fig. 1

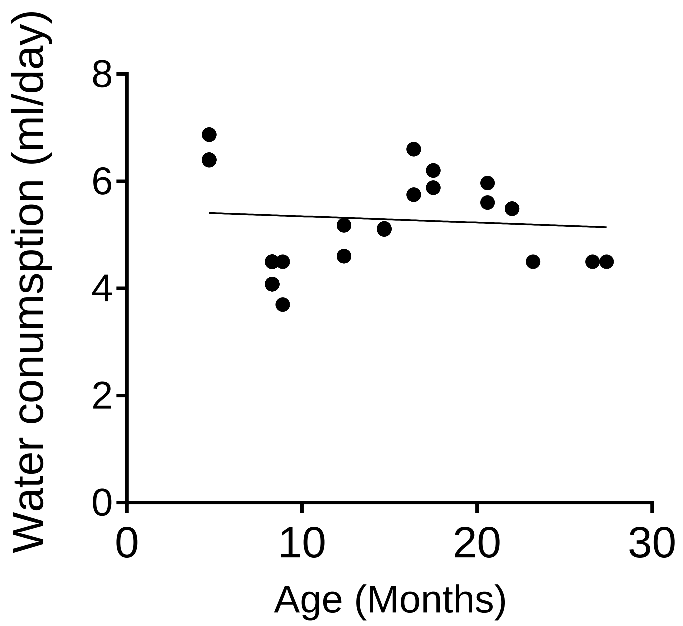

Supplemental Fig. 2

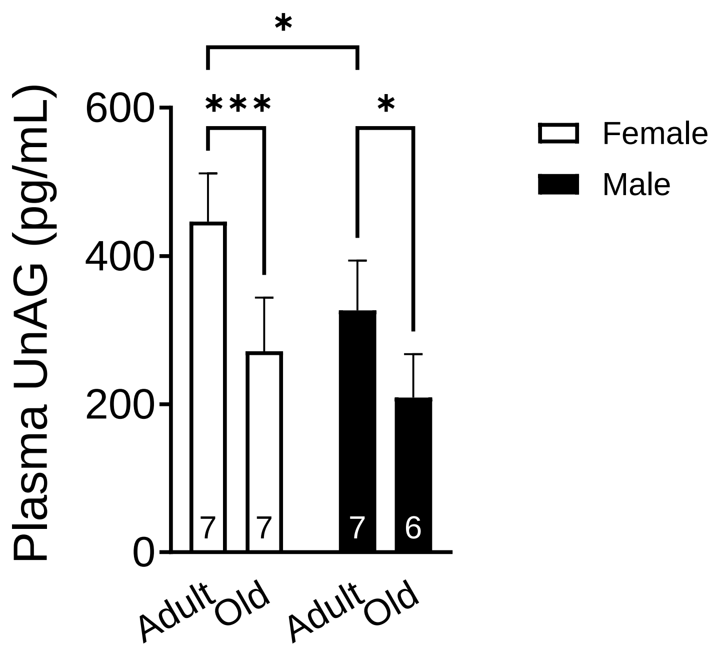

Supplemental Fig. 3

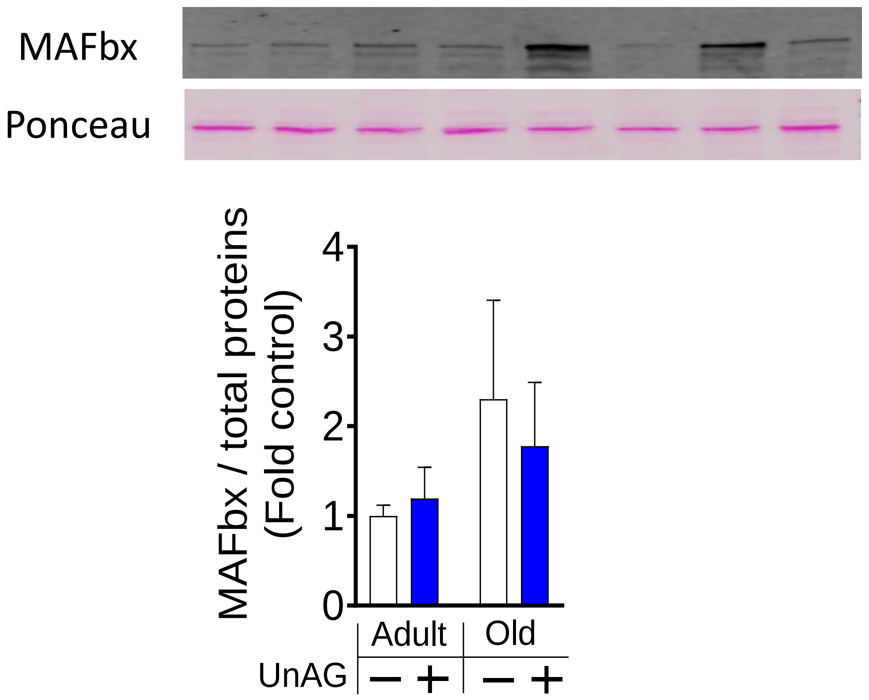

Supplemental Fig. 4

H_2O_2 standard assay

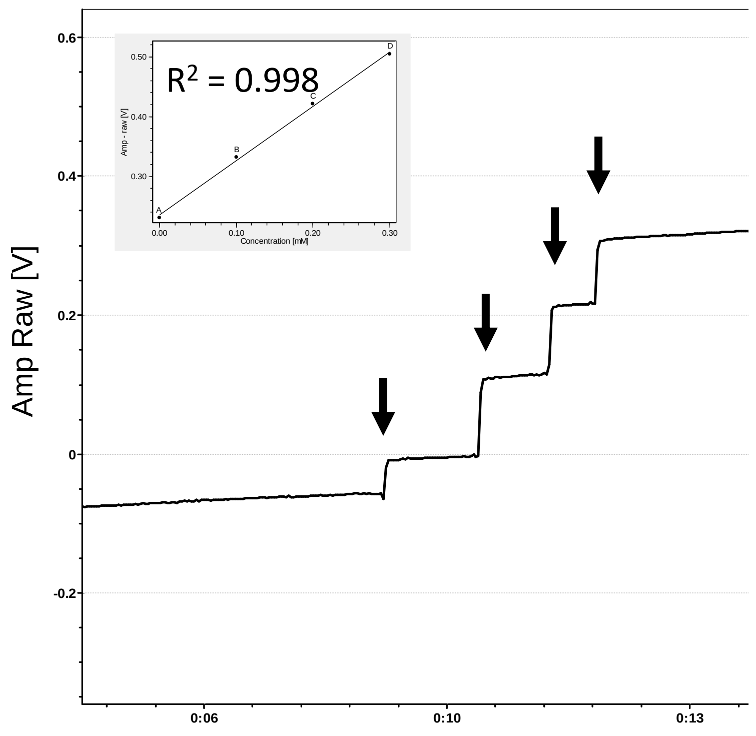

Supplemental Fig. 5

(A) **Primary Antibodies used for immunoblots**

Primary antibodies	Company, catalogue #	Ratio, incubation
MuRF1	SCBT, sc-398608	1:500, overnight
MAFbx	SCBT, sc-166806	1:1000, overnight
mTOR	Genetex, GTX101557	1:1000, overnight

(B) qRT-PCR Primer Sequences

Gene Name	Forward Primer	Reverse Primer
AchR-a	ACCTGGACCTATGACGGCTCT	AGTTACTCAGGTCGGGCTGGT
GADD45a	AGACCGAAAGGATGGACACG	GTACACGCCGACCGTAATG
Runx1	GATGGCACTCTGGTCACCG	GCCGCTCGGAAAAGGACAA

Supplemental Table.
